# Elephantiasis of the Clitoris

**Published:** 1860-04

**Authors:** 


*Elephantiasis of the Clitoris.*—A well marked example of this curious disease, spreading to the surrounding labia and nymphæ, was recently admitted into St. George's Hospital. The patient was an elderly woman, who underwent an operation three years ago for the removal of the same sort of disease, but it had not now returned in the old cicatrix. It formed a considerable sized tumor of the organ in question, was pendulous, and divided into a series of lobes by numerous fringe-like processes. She was placed under the influence of chloroform, and its removal was effected by means of a double ligature through its pedicle, and then cutting it off. This totally prevented any hemorrhage, which Mr. Tatum observed was some times very considerable in such cases. The patient is doing well, but much disease remains in the labia and nymphæ, the cellular tissue of which is very liable to take on the same hypertrophic action of a later period.

